# A generalized eigenvector centrality for multilayer networks with inter-layer constraints on adjacent node importance

**DOI:** 10.1007/s41109-024-00620-8

**Published:** 2024-04-30

**Authors:** H. Robert Frost

**Affiliations:** Dartmouth College, Hanover, NH 03755 USA

**Keywords:** Eigenvector centrality, Multilayer networks, Power iteration

## Abstract

We present a novel approach for computing a variant of eigenvector centrality for multilayer networks with inter-layer constraints on node importance. Specifically, we consider a multilayer network defined by multiple edge-weighted, potentially directed, graphs over the same set of nodes with each graph representing one layer of the network and no inter-layer edges. As in the standard eigenvector centrality construction, the importance of each node in a given layer is based on the weighted sum of the importance of adjacent nodes in that same layer. Unlike standard eigenvector centrality, we assume that the adjacency relationship and the importance of adjacent nodes may be based on distinct layers. Importantly, this type of centrality constraint is only partially supported by existing frameworks for multilayer eigenvector centrality that use edges between nodes in different layers to capture inter-layer dependencies. For our model, constrained, layer-specific eigenvector centrality values are defined by a system of independent eigenvalue problems and dependent pseudo-eigenvalue problems, whose solution can be efficiently realized using an interleaved power iteration algorithm. We refer to this model, and the associated algorithm, as the Constrained Multilayer Centrality (CMLC) method. The characteristics of this approach, and of standard techniques based on inter-layer edges, are demonstrated on both a simple multilayer network and on a range of random graph models. An R package implementing the CMLC method along with example vignettes is available at https://hrfrost.host.dartmouth.edu/CMLC/.

## Background

### Eigenvector centrality

Computation of node importance via centrality measures is an important task in network analysis and a large number of centrality measures have been developed that prioritize different node and network properties (Newman 2010). The most widely used centrality measures are a function of the network adjacency matrix, ** A**, which, for an edge-weighted network defined over *p* nodes is the *p* × *p* matrix:

1 $$\begin{aligned} {\mathbf{A}} = \begin{bmatrix} a_{1,1} &{}\quad \cdots &{}\quad a_{1,p} \\ \vdots &{}\quad \ddots &{}\quad \vdots \\ a_{p,1} &{}\quad \cdots &{}\quad a_{p,p} \end{bmatrix} \end{aligned}$$

where *a*_*i*,*j*_ captures the weight of the edge between nodes *i* and *j* or 0 if no edge exists between these nodes. Self-edges are represented by elements on the diagonal. If the network is directed, then *a*_*i*,*j*_ and *a*_*j*,*i*_ capture distinct edges and ** A** is asymmetric; if the network is undirected, $a_{i,j} = a_{j,i}$ and ** A** is symmetric.

Modeling node importance as the weighted sum of the importance of adjacent nodes leads a version of centrality called eigenvector centrality, which is solved by computing the principal eigenvector of the following eigenvalue problem:

2 $$\begin{aligned} {\mathbf{A}} {\mathbf{x}} = \lambda {\mathbf{x}} \end{aligned}$$

Specifically, the eigenvector centrality for node *i* is given by element *i* of the principal eigenvector ${\mathbf{x}}$ corresponding to the largest eigenvalue (Newman 2010). When ** A** is irreducible (i.e., the network is strongly connected), then the Perron–Frobenius theorem (Perron 1907) guarantees that there is a unique largest real eigenvalue whose corresponding eigenvector can be chosen to have strictly positive elements. For directed graphs, left and right versions of eigenvector centrality are possible, i.e., the solution to the eigenvalue problem for ${\mathbf{A}}^T$ or ** A**. For the methods developed below, we focus on the right eigenvector centrality, however, the same approach can be employed to compute left eigenvector centrality by considering ${\mathbf{A}}^T$ instead of ** A**.

### Multilayer eigenvector centrality

In this paper, we are interested in eigenvector centrality and how that measure of node importance generalizes to multilayer (or multiplex) networks (Battiston et al. 2014; Kivelä et al. 2014). We assume a multilayer network comprised by *k* layers that each represent a potentially directed, edge-weighted graph over the same *p* nodes. The graph for layer $j \in \{1, \ldots ,k\}$ can be represented by the adjacency matrix ${\mathbf{A}}_j$:

3 $$\begin{aligned} {\mathbf{A}}_j = \begin{bmatrix} a_{j,1,1} &{} \cdots &{} a_{j,1,p} \\ \vdots &{} \ddots &{} \vdots \\ a_{j,p,1} &{} \cdots &{} a_{j,p,p} \end{bmatrix} \end{aligned}$$

where $a_{j,l,m}$ holds the weight of the edge from node *l* to node *m* within the layer *j* graph. Although the terms network and graph are synonymous in this context, we will generally use the term network to refer to the entire multilayer network and the term graph to refer to the network that defines a single layer. In the context of a multilayer network, node eigenvector centrality can be evaluated at the level of a specific layer (i.e., a given node has separate centrality values for each of the *k* layers) or at the level of the entire multilayer network (i.e., a given node has a single centrality value that captures the importance of the node across all *k* layers). In the development below, we focus on layer-specific measures of eigenvector centrality. If the *k* layers are independent, then eigenvector centrality can simply be computed separately for each layer. However, if dependencies exist between the layers, then a multilayer version of eigenvector centrality must be employed that can account for the inter-layer constraints.

### Existing approaches for multilayer eigenvector centrality

A number of approaches for modeling and computing multilayer eigenvector centrality have been explored over the last decade (e.g., Tudisco et al. 2018; Taylor et al 2021; De Domenico et al. 2015; DeFord and Pauls 2018; Solà et al. 2013; Kumar et al. 2023). Most of these approaches assume that inter-layer constraints can be modeled by edges between the nodes in one layer and nodes in other layers. This type of approach is exemplified by the recent work of Taylor et al (2021) that details a flexible model for a “uniformly and diagonally coupled multiplex network”. Specifically, Taylor et al. represent inter-layer dependencies by equally weighted edges connecting the nodes in one layer to the same nodes in a dependent layer. More flexible models based on inter-layer edges, such as MultiCens (Kumar et al. 2023), support inter-layer edges between any pair of nodes. Taylor et al. represent the structure of these dependencies using a $k \times k$ adjacency matrix $\tilde{{\mathbf{A}}}$:

4 $$\begin{aligned} \tilde{{\mathbf{A}}} = \begin{bmatrix} {\tilde{a}}_{1,1} &{}\quad \cdots &{}\quad {\tilde{a}}_{1,k} \\ \vdots &{}\quad \ddots &{}\quad \vdots \\ {\tilde{a}}_{k,1} &{}\quad \cdots &{}\quad {\tilde{a}}_{k,k} \end{bmatrix} \end{aligned}$$

where ${\tilde{a}}_{i,j}$ represents the weight of the edges from nodes in layer *i* to nodes in layer *j*. Computation of multilayer eigenvector centralities is then based on the principal eigenvector of a $kp \times kp$ supercentrality matrix ${\mathbb {C}}(\omega )$:

5 $$\begin{aligned} {\mathbb {C}}(\omega ) = \hat{{\mathbb {C}}} + \omega \hat{{\mathbb {A}}} \end{aligned}$$

where $\hat{{\mathbb {C}}} = diag[{\mathbf{A}}_1, \ldots , {\mathbf{A}}_k]$ (i.e., a $kp \times kp$ block diagonal matrix that has the adjacency matrices for each of the *k* layers along the diagonal), $\hat{{\mathbb {A}}} = \tilde{{\mathbf{A}}} \otimes {\mathbf{I}}$ (i.e., the Kronecker product of $\tilde{{\mathbf{A}}}$ and ${\mathbf{I}}$), and $\omega$ is the coupling strength. The principal eigenvector of ${\mathbb {C}}(\omega )$ can then be used to find joint, marginal, and conditional eigenvector centralities. Specifically, the principal eigenvector elements are divided into *k* sequential blocks of *p* elements, with the block corresponding to layer *i* representing the joint centrality values for the nodes in layer *i*. To calculate the marginal centralities for either nodes or layers, the joint centralities are summed over all layers for a given node or all nodes for a given layer. To calculate conditional centralities for either nodes or layers, the joint centrality value for a given node/layer pair is divided by either the marginal centrality for the layer or the marginal centrality for the node.

### Distinguishing between node adjacency and adjacent node importance

Approaches like Taylor et al. that use inter-layer edges to capture dependencies between layers effectively turn a multi-layer network into a single large interconnected graph. While this approach is appropriate and effective in many scenarios, there is a specific use case that this model fails to support. In particular, we are interested in the scenario where the centrality of a given node in one layer is based on the weighted sum of the importance of adjacent nodes (as defined by the topology of that layer) but with adjacent node importance potentially based on other layers. We believe this type inter-layer dependency model (which we define more specifically in the “Eigenvector centrality for multilayer networks with inter-layer constraints on adjacent node importance” section below) has utility for a number of important real world multilayer network analysis problems where node adjacency and adjacent node importance are captured by distinct networks, e.g., transportation networks.

One specific example of such a real world problem involves the characterization of ligand/receptor mediated cell-cell communication within a tissue. This cell signaling problem was in fact the original motivation for this paper. In this scenario, we assume that each cell is one of several distinct cell types, e.g., CD8^+^ T cell, and that each cell type is capable of presenting a set of membrane-bound receptor proteins (e.g., PD1 for CD8^+^ T cells) on its surface with the set of receptors associated with different cell types potentially overlapping. We additionally assume that each receptor has a unique cognate secreted ligand protein (e.g., PDL1 for PD1) that can bind to it and that each ligand is produced as a consequence of one or more receptor signaling pathways, i.e., binding of a given receptor by its associated ligand will trigger production of other ligands by the cell.

Given this simple ligand/receptor signaling model and the distribution of cells within a tissue, a key question is to estimate the steady-state activity of each receptor signaling pathway. One approach for answering that question creates a multilayer network with one directed layer per receptor protein. The layer for a specific receptor would be fully connected with nodes representing cells and directed edges pointing into each node whose weight is either the inverse squared distance between the source and target cells when the target cell type expresses the receptor or a small default value if the cell type does not express the receptor (this small value ensures the network is fully connected). Ideally, the activity of a specific receptor signaling pathway in a given cell would be represented by the centrality of the associated node in the layer corresponding to that receptor. However, simply computing the eigenvector centrality for each layer does not yield the appropriate answer since the importance of adjacent cells in a given receptor layer reflects that activity of that receptor, which most likely does not impact the activity of that same receptor in other cells, i.e., binding of a given receptor does in general result in secretion of the associated ligand. Instead, one wants to use the importance of cells in the layers corresponding to receptors that produce the cognate ligand. This type of inter-layer dependency structure is exactly what the model we outline in the “Eigenvector centrality for multilayer networks with inter-layer constraints on adjacent node importance” section supports. We believe that a more general class of systems biology questions may map to similar interdependent multilayer networks.

### Updates relative to complex networks manuscript

This manuscript extends an earlier version of the paper published in the Proceedings of the 12th International Conference on Complex Networks and their Applications (Frost 2023). In particular, the following non-trivial updates have been made relative to the conference proceedings paper:

- *Comparison against multilayer eigenvector centrality approach that uses inter-layer edges* Results from a multilayer eigenvector centrality method based on inter-layer edges that is similar to the approach of Taylor et al. are now included in the results for the simple and random example networks (“Simple example”, “Random graph example using inter-layer edges” sections) and in the expanded simulation study in the “Simulation analysis” section. The implementation of the comparison method is detailed in the “Comparison method based on inter-layer edges” section.
- *Expanded simulation study* To more thoroughly characterize the behavior of Algorithm 1, an expanded simulation study has been added as the “Simulation analysis” section. Specifically, Algorithm 1 and the comparison inter-layer edge approach were used to compute eigenvector centrality values for simulated multilayer networks with a variable number of layers and number of nodes per layer. For each simulated network structure, the number of iterations to convergence for Algorithm 1, the relative execution time of Algorithm 1 and the inter-layer edge approach and the difference in centrality values were computed and are visualized in Figs. 8, 9, and 10.
- *Negative inter-layer dependencies* The proposed multilayer eigenvector centrality approach has been modified to support both positive and negative inter-layer dependency weights, i.e., elements of the inter-layer dependency adjacency matrix $\tilde{{\mathbf{A}}}$ defined in (10). In the original version, the dependency weights for a single layer were constrained to be positive and to sum to 1. This constraint has been relaxed to allow for some weights to be negative as long as the sum of all weights for a given layer is 1. While negatively weighted dependencies enable a number of interesting scenarios, it is important to note that this type of dependency structure can result in negative centrality values and may have a very slow rate of convergence so appropriate cautionary language has been added to the method text (and the R method also issues a warning in this scenario). The use of negative weights has been intergrated into the simple and random network examples in the “Simple example” and “Random graph example using negative dependency weights” sections.
- *Per-layer convergence threshold* The original version of Algorithm 1 determined convergence based on the mean sequential difference in relative eigenvalues (or pseudo-eigenvalues) across all layers. If $\lambda _{j,i}$ is the estimate of the largest eigenvalue for layer *j* after iteration *i*, then the relative sequential difference for layer *j* after iteration *i* is given by $\delta _{j,i} = (|\lambda _{j, i-1} - \lambda _{j,i}|)/\lambda _{j,i}$ and convegence is assumed when $1/k \sum _{j=1}^{k} \delta _{j,i} <= tol$, where *tol* is a customizable parameter. The updated version of the method now allows convergence to be optionally assessed for each layer independently with overall convergence only assumed when each layer has converged. This change guards against the scenario where the algorithm terminates when a small number of layers are still far from convergence.

## Methods

### Eigenvector centrality for multilayer networks with inter-layer constraints on adjacent node importance

Our model for multilayer eigenvector centrality extends the standard single network version given by (2) to support the scenario where the importance of a given node in layer *i* is proportional to the weighted sum of the importance of adjacent nodes with adjacency and weights based on layer *i* but adjacent node importance based on potentially distinct layers. To restate this in the context of the ligand/receptor example outlined in the “Distinguishing between node adjacency and adjacent node importance” section, this model has the interpretation that the activity of a given receptor, say PD1, in each cell is based on the activity in adjacent cells of receptors that generate the ligand for this receptor (i.e., PDL1). Because the activity of each receptor is represented by a distinct layer for this example, this model separates node adjacency and adjacent node importance across potentially distinct layers. We will refer to this model and the associated algorithm (see Algorithm 1 below) as Constrained Multilayer Centrality (CMLC). This model is conceptually and mathematically distinct from approaches like Taylor et al. that add edges between the same node in different layers to capture inter-layer dependencies. To illustrate, assume we have a multilayer network with just two layers *i* and *j*. If we assume the importance for nodes in layer *i* has the standard definition, i.e., it is not dependent on another layer, the solution is given by the typical eigenvalue problem:

6 $$\begin{aligned} {\mathbf{A}}_i {\mathbf{x}}_i = \lambda _i {\mathbf{x}}_i \end{aligned}$$

However, if we assume the importance for nodes in layer *j* is based on the importance of adjacent nodes in layer *i*, then the solution for layer *j* is given by the following linear model (note that both ${\mathbf{x}}_i$ and ${\mathbf{x}}_j$ are included):

7 $$\begin{aligned} {\mathbf{A}}_j {\mathbf{x}}_i = \lambda _j {\mathbf{x}}_j \end{aligned}$$

Although the linear model (7) is not technically an eigenvalue problem since it contains distinct ${\mathbf{x}}_i$ and ${\mathbf{x}}_j$ vectors, we will refer to it as a pseudo-eigenvalue problem given the structural similarities to (6) and the fact that ${\mathbf{x}}_i$ may represent an eigenvector. As detailed below, we will also use the pseudo-eigenvalue label to describe more complex scenarios where ${\mathbf{x}}_i$ is found on both sides of the equation. For this example, the solution for the entire multilayer network is given by a system of an independent eigenvalue problem and a dependent pseudo-eigenvalue problem:

8 $$\begin{aligned} \begin{array}{rcl} {\textbf{A}}_i {\textbf{x}}_i &{}= \lambda _i {\textbf{x}}_i \\ {\textbf{A}}_j {\textbf{x}}_i &{}= \lambda _j {\textbf{x}}_j \end{array} \end{aligned}$$

In this case, the solution can be obtained by first solving the eigenvalue problem for layer *i* to find ${\textbf{x}}_i$ and then computing ${\textbf{x}}_j$ as ${\textbf{x}}_j = 1/\lambda _j {\textbf{A}}_j {\textbf{x}}_i$ with the value of $\lambda _j$ set to ensure ${\textbf{x}}_j$ is unit length. Importantly, the supercentrality approach of Taylor et al. cannot directly solve systems such as (8). This can be illustrated by rewriting (8) as a single linear model:

9 $$\begin{aligned} \begin{bmatrix} {\textbf{A}}_i \\ {\textbf{A}}_j \end{bmatrix} {\textbf{x}}_i = \begin{bmatrix} \lambda _i {\textbf{x}}_i \\ \lambda _j {\textbf{x}}_j \end{bmatrix} \end{aligned}$$

which cannot be exactly mapped to an eigenvalue problem involving a supercentrality matrix of the form defined by (5).

This simple example can be generalized to a multilayer network with *k* layers and arbitrary node importance constraints encoded by a graph whose nodes represent layers and whose weighted and directed edges represent inter-layer dependencies. Let this inter-layer dependency graph be represented by the $k \times k$ adjacency matrix $\tilde{{\textbf{A}}}$ that is similar in structure to the $\tilde{{\textbf{A}}}$ used by Taylor et al. and defined in (4). but with the added constraint that the rows must sum to 1 (i.e., $\forall _{i \in 1,\ldots ,k} \sum _{j=1}^{k} {\tilde{a}}_{i,j} = 1$). Element ${\tilde{a}}_{i,j}$ of $\tilde{{\textbf{A}}}$ represents the strength of the dependency between adjacent node importance in layer *i* and node importance in layer *j*. It is important to note that negative values are allowed in $\tilde{{\textbf{A}}}$ as long as the sum of all dependencies for a layer equals one. Although the use of negative weights in $\tilde{{\textbf{A}}}$ enables the modeling of inverse dependencies between the importance of nodes in one layer and the importance of adjacent nodes in other layers, it can lead to negative centrality values and impact the rate of convergence.

If $\tilde{{\textbf{A}}} = {\textbf{I}}$, all of the layers are independent. For the 2 layer example represented by (8), $\tilde{{\textbf{A}}}$ is:

10 $$\begin{aligned} \tilde{{\textbf{A}}} = \begin{bmatrix} 1 &{}\quad 0 \\ 1 &{}\quad 0 \end{bmatrix} \end{aligned}$$

If the length *p* vector ${\textbf{x}}_i$ represents node importance in layer *i*, we can define an adjacent node importance function ${\textbf{c}}(i,\tilde{{\textbf{A}}})$ as:

11 $$\begin{aligned} {\textbf{c}}(i, \tilde{{\textbf{A}}}) = \sum _{j=1}^{k} {\tilde{a}}_{i,j} {\textbf{x}}_j \end{aligned}$$

In other words, the importance of nodes in layer *i* is based on a weighted sum of the importance of adjacent nodes in other layers (note that adjacency is only based on the topology of layer *i*). Given the function ${\textbf{c}}()$, we can compute a constrained multilayer version of eigenvector centrality for the network by solving the following system of *k* interdependent eigenvalue and pseudo-eigenvalue problems:

12 $$\begin{aligned} \begin{array}{rcl} {\textbf{A}}_1 {\textbf{c}}(1, \tilde{{\textbf{A}}} ) &{}= \lambda _1 {\textbf{x}}_1 \\ {\textbf{A}}_2 {\textbf{c}}(2, \tilde{{\textbf{A}}}) &{}= \lambda _2 {\textbf{x}}_2 \\ &{}\vdots \\ {\textbf{A}}_k {\textbf{c}}(k, \tilde{{\textbf{A}}}) &{}= \lambda _k {\textbf{x}}_k \end{array} \end{aligned}$$

Importantly, the supercentrality approach of Taylor et al. cannot in general solve systems such as (8), i.e., system (12) cannot be directly mapped to an eigenvalue problem involving a supercentrality matrix of the form defined by (5). Specifically, an approach based on introduced edges between layers can never fully decouple the adjacency relationship from the importance of adjacent nodes, e.g., the model where one layer is fully dependent on another layer (inter-layer dependency matrix in “Simple example” section) is impossible to capture using inter-layer edges.

In the special case where $\tilde{{\textbf{A}}} = {\textbf{I}}$, all ${\textbf{c}}(i, \tilde{{\textbf{A}}}) = {\textbf{x}}_i$ and (12) becomes a system of *k* independent eigenvalue problems:

13 $$\begin{aligned} \begin{array}{rcl} {\textbf{A}}_1 {\textbf{x}}_1 &{}= \lambda _1 {\textbf{x}}_1 \\ {\textbf{A}}_2 {\textbf{x}}_2 &{}= \lambda _2 {\textbf{x}}_2 \\ &{}\vdots \\ {\textbf{A}}_k {\textbf{x}}_k &{}= \lambda _k {\textbf{x}}_k \\ \end{array} \end{aligned}$$

More generally, the dependency structure for a given layer *i* falls into one of three cases:

A ${\tilde{a}}_{i,i} = 1$. In this scenario, the constraint that each row of $\tilde{{\textbf{A}}}$ sums to 1 means that all other entries in the *i*th row are 0, i.e., $\forall _{j \ne i} {\tilde{a}}_{i,j} = 0$. This implies that the eigenvector centrality for layer *i* is given by the principal eigenvector of the independent eigenvalue problem ${\textbf{A}}_i {\textbf{x}}_i = \lambda _i {\textbf{x}}_i$.
B ${\tilde{a}}_{i,i} = 0$: In this scenario, the centrality for layer *i* is a linear function of the centrality values of other network layers.
C ${\tilde{a}}_{i,i} \ne 0 {\text{ and }} {\tilde{a}}_{i,i} \ne 1$: In this scenario, the centrality for layer *i* is given by a pseudo-eigenvalue problem that can be rewritten as ${\textbf{A}}_i {\textbf{x}}_i + {\textbf{d}} = \lambda _i {\textbf{x}}_i$, where ${\textbf{d}}$ is captures the part of ${\textbf{A}}_i {\textbf{c}}(i, \tilde{{\textbf{A}}} )$ not due to ${\textbf{x}}_i$. While this pseudo-eigenvalue formulation is structurally similar to the PageRank centrality equation (Brin and Page 1998), the ${\textbf{d}}$ term on the left-hand side is not a fixed constant but is instead dependent on the solutions of the equations associated with other layers in (12).

If all layers in the multilayer network fall into case A or B and no cycles exist in the graph defined by $\tilde{{\textbf{A}}}$, then the constrained eigenvector centralities can be computed using a relatively straightforward two-step procedure:

1. Solve the independent eigenvalue problems for all layers in case A using an algorithm like power iteration (von Mises and Pollaczek-Geiringer 1929).
2. Sequentially solve the linear models for all layers in case B with the order of solution given by the inter-layer constraints.

If any layers fall into case C or cycles exist in the inter-layer dependency graph, then the solution must be obtained via an iterative algorithm similar to the interleaved power iteration approach detailed in the “Interleaved power iteration algorithm for a system of dependent pseudo-eigenvalue problems” section as Algorithm 1.

### Interleaved power iteration algorithm for a system of dependent pseudo-eigenvalue problems

For an arbitrary inter-layer dependency matrix $\tilde{{\textbf{A}}}$, the joint solution for system (12) can be found via a interleaved version of the power iteration method, detailed in Algorithm 1, that is applied across all *k* linear problems. It should be noted that this specification of Algorithm 1 does not include features important for many practical implementations, e.g, checks to ensure the input matrices ${\textbf{X}}_i$ are well conditioned, options for the use of stochastic initialization of the eigenvectors, use of techniques like accelerated stochastic power iteration (Xu et al. 2018) to improve computational performance, alternate stopping conditions, etc..

**Algorithm 1:**
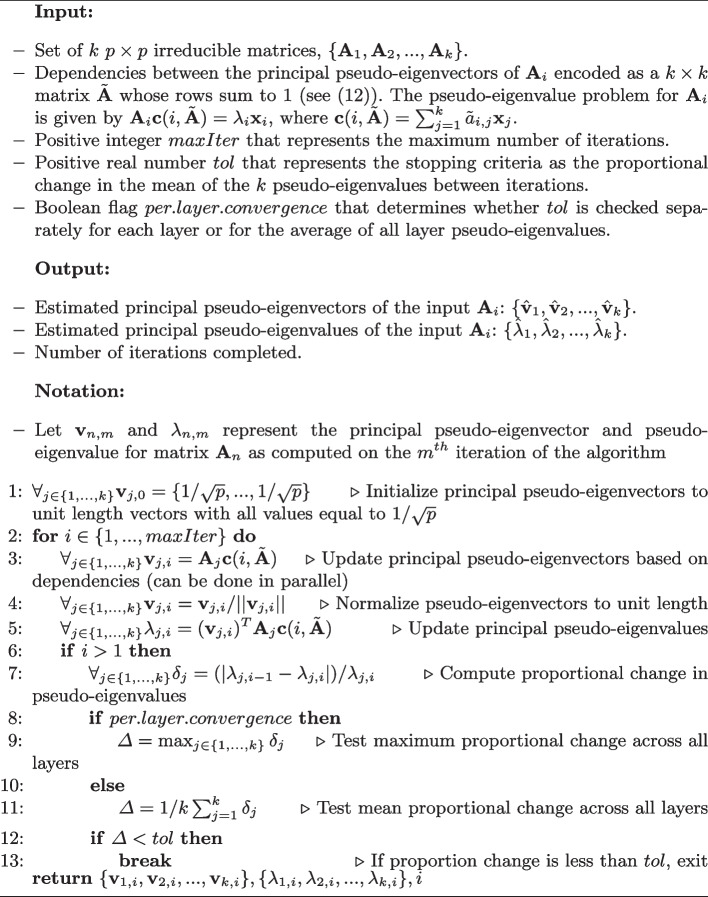
Interleaved power iteration for dependent pseudo-eigenvalue problems

### Comparison method based on inter-layer edges

To contrast the performance of the approach detailed Algorithm 1 against existing multilayer eigenvector centrality approaches based on inter-layer edges (e.g., the technique of Taylor et al (2021) detailed in the “Existing approaches for multilayer eigenvector centrality” section above), we have implemented an inter-layer edge method in the CMLC R package. This technique uses the inter-layer dependencies defined in $\tilde{{\textbf{A}}}$ to create a single large network through the introduction of inter-layer edges. Specifically, if the off-diagonal element ${\tilde{a}}_{i,j}$ of $\tilde{{\textbf{A}}}$ is non-zero, then equal-weighted directed edges are introduced between all nodes in layer *i* and the same nodes in layer *j*. Standard eigenvector centrality is then computed for this single network using the power iteration technique with the centrality values for each specific layer normalized to length 1. For this implementation, the adjacency matrix for the combined network is represented in the standard dense format rather than a sparse format such as that supported by the *Matrix* R package. This is equivalent to using the normalized joint centrality values computed via the Taylor et al. approach with coupling strength parameter $\omega =1$ and zero entries on the diagonal of $\tilde{{\textbf{A}}}$. Results from this comparative approach have been added to the examples in the “Simple example” and “Random graph example” sections.

### CMLC R package

An implementation of Algorithm 1 and the comparison inter-layer edge technique described in the “Comparison method based on inter-layer edges” section are available via the CMLC (Constrained Multilayer Centrality) R package. The CMLC R package along with example vignettes that generate the results in the “Simple example” to “Simulation analysis” sections is available at https://hrfrost.host.dartmouth.edu/CMLC/.

## Results

### Simple example

**Fig. 1:**
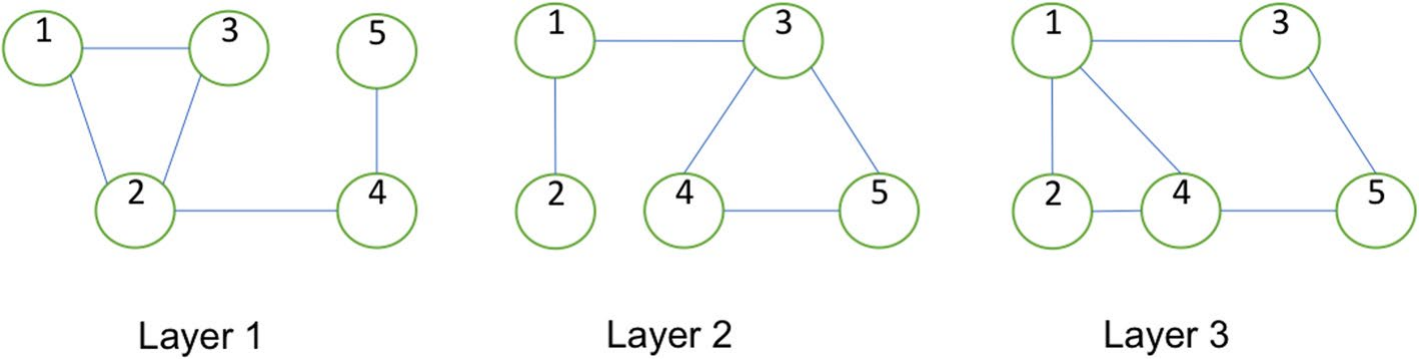
Example undirected and unweighted multilayer network

To illustrate the constrained multilayer model detailed in the “Eigenvector centrality for multilayer networks with inter-layer constraints on adjacent node importance” section and the performance of the interleaved power iteration algorithm detailed in the “Interleaved power iteration algorithm for a system of dependent pseudo-eigenvalue problems” section, we consider a simple multilayer network comprised by three layers that each define an undirected and non-weighted network with five nodes. The structure of this multilayer network is shown in Fig. 1.

For this example network, the symmetric adjacency matrices for the three layers are given by:

$$\begin{aligned} \begin{array}{rcl} {\textbf{A}}_1 &{}= \begin{bmatrix} 0 &{}\quad 1 &{}\quad 1 &{}\quad 0 &{}\quad 0 \\ 1 &{}\quad 0 &{}\quad 1 &{}\quad 1 &{}\quad 0 \\ 1 &{}\quad 1 &{}\quad 0 &{}\quad 0 &{}\quad 0 \\ 0 &{}\quad 1 &{}\quad 0 &{}\quad 0 &{}\quad 1 \\ 0 &{}\quad 0 &{}\quad 0 &{}\quad 1 &{}\quad 0 \end{bmatrix}, {\textbf{A}}_2 = \begin{bmatrix} 0 &{}\quad 1 &{}\quad 1 &{}\quad 0 &{}\quad 0 \\ 1 &{}\quad 0 &{}\quad 0 &{}\quad 0 &{}\quad 0 \\ 1 &{}\quad 0 &{}\quad 0 &{}\quad 1 &{}\quad 1 \\ 0 &{}\quad 0 &{}\quad 1 &{}\quad 0 &{}\quad 1 \\ 0 &{}\quad 0 &{}\quad 1 &{}\quad 1 &{}\quad 0 \end{bmatrix}, {\textbf{A}}_3 = \begin{bmatrix} 0 &{}\quad 1 &{}\quad 1 &{}\quad 1 &{}\quad 0 \\ 1 &{}\quad 0 &{}\quad 0 &{}\quad 1 &{}\quad 0 \\ 1 &{}\quad 0 &{}\quad 0 &{}\quad 0 &{}\quad 1 \\ 1 &{}\quad 1 &{}\quad 0 &{}\quad 0 &{}\quad 1 \\ 0 &{}\quad 0 &{}\quad 1 &{}\quad 1 &{}\quad 0 \end{bmatrix} \\ \end{array} \end{aligned}$$

We consider five different inter-layer dependency scenarios:

1. *No dependencies* If no dependencies exist between the layers (i.e., $\tilde{{\textbf{A}}}= {\textbf{I}}$), the eigenvector centralities (rounded to two decimal places) for each layer are:
  $$\begin{aligned} \begin{array}{rcl} {\textbf{v}}_1 &{}= \{0.50,0.60,0.50,0.34,0.15\}\\ {\textbf{v}}_2 &{}= \{0.34,0.15,0.60,0.50,0.50\} \\ {\textbf{v}}_3 &{}= \{0.53, 0.43, 0.36, 0.53, 0.36\} \end{array} \end{aligned}$$
  As expected given the structure of layer 1, node 2 has the largest eigenvector centrality, followed by nodes 1 and 3 with node 5 having the lowest. Similarly for layer 2, node 3 has the largest centrality, followed by nodes 4 and 5 with node 2 having the lowest centrality. For layer 3, nodes 1 and 4 are tied for the largest centrality with nodes 3 and 5 tied for the lowest.
2. *Mixture of layer dependency cases A and B* If layer 1 is independent, layer 2 is dependent on just layer 1 and layer 3 is dependent on layer 2, the $\tilde{{\textbf{A}}}$ matrix takes the form:
  $$\begin{aligned} \tilde{{\textbf{A}}} = \begin{bmatrix} 1 &{} 0 &{} 0 \\ 1 &{} 0 &{} 0 \\ 0 &{} 1 &{} 0 \end{bmatrix} \end{aligned}$$
  and the constrained eigenvector centralities are:
  $$\begin{aligned} \begin{array}{rcl} {\textbf{v}}_1 &{}= \{0.50,0.60,0.50,0.34,0.15\}\\ {\textbf{v}}_2 &{}= \{0.58,0.26,0.53,0.34,0.44\} \\ {\textbf{v}}_3 &{}= \{0.48, 0.39, 0.43, 0.54, 0.37\} \end{array} \end{aligned}$$
  Since layer 1 is still independent in this scenario, it has the same centrality values as the prior case. For layer 2, we see the expected increase in the centrality of node 1 relative to node 3 given the importance of their adjacent nodes in layer 1 (i.e, node 1 is adjacent to node 2, which has the largest centrality value in layer 1; node 3 is adjacent to nodes 4 and 5, which have the lowest centrality values in layer 1). For layer 3, the centrality for node 3 has the largest change (an increase) relative to the independent scenario, which is expected given that it is adjacent to the node with the largest centrality value in layer 2 (node 1). To illustrate the differences between our proposed technique and standard approaches for multilayer eigenvector centrality, the centrality values for this case were also computed using the comparison method outlined in the “Comparison method based on inter-layer edges” section that represents dependencies between layers using inter-layer edges. For this dependency scenario, this is equivalent to all of the nodes in layer 2 having directed edges to the same nodes in layer 1 with weight 1. After introduction of these edges, the entire multilayer network can be represented by a single network with *pk* nodes and a $pk \times pk$ adjacency matrix defined according to (5) with coupling strength parameter $\omega =1$. The multilayer eigenvector centrality values can then be computed using the normal eigenvector centrality formulation on the merged network, which results in:
  $$\begin{aligned} \begin{array}{rcl} {\textbf{v}}_1 &{}= \{0.54,0.50,0.48,0.46,0.14\}\\ {\textbf{v}}_2 &{}= \{0.39,0.24,0.60,0.47,0.46\} \\ {\textbf{v}}_3 &{}= \{0.53,0.43,0.36,0.53,0.36\} \end{array} \end{aligned}$$
  This type of approach obviously has a very different mathematical interpretation from an approach which uses adjacent node importance to capture inter-layer dependencies and, as expected, the constrained centrality values are distinct from those generated according to the proposed adjacent node importance technique defined by Algorithm 1.
3. *Mixture of layer dependency cases A, B and C* If layer 1 is independent, layer 2 is dependent on just layer 1 and layer 3 is equally dependent on both layer 2 and itself, the $\tilde{{\textbf{A}}}$ matrix takes the form:
  $$\begin{aligned} \tilde{{\textbf{A}}} = \begin{bmatrix} 1 &{}\quad 0 &{}\quad 0 \\ 1 &{}\quad 0 &{}\quad 0 \\ 0 &{}\quad 0.5 &{}\quad 0.5 \end{bmatrix} \end{aligned}$$
  and the constrained eigenvector centralities are:
  $$\begin{aligned} \begin{array}{rcl} {\textbf{v}}_1 &{}= \{0.50,0.60,0.50,0.34,0.15\}\\ {\textbf{v}}_2 &{}= \{0.58,0.26,0.53,0.34,0.44\} \\ {\textbf{v}}_3 &{}= \{0.51,0.41,0.39,0.53,0.37\} \end{array} \end{aligned}$$
  Since layers 1 and 2 have the same dependency structure as the prior scenario, the centrality values are unchanged. As expected, the equally divided dependency structure for layer 3 yields centrality values that are between those computed in the first two scenarios.
4. *Mixture of layer dependency cases A, B and C with negative dependency* To illustrate the use of negative dependency weights, we explored the case where layer 1 is independent, layer 2 is dependent on just layer 1 and layer 3 has a positive dependence on itself and a small negative dependency on layer 2, the $\tilde{{\textbf{A}}}$ matrix takes the form:
  $$\begin{aligned} \tilde{{\textbf{A}}} = \begin{bmatrix} 1 &{}\quad 0 &{}\quad 0 \\ 1 &{}\quad 0 &{}\quad 0 \\ 0 &{}\quad -\,0.2 &{}\quad 1.2 \end{bmatrix} \end{aligned}$$
  The constrained eigenvector centrality values for this scenario are
  $$\begin{aligned} \begin{array}{rcl} {\textbf{v}}_1 &{}= \{0.50,0.60,0.50,0.34,0.15\}\\ {\textbf{v}}_2 &{}= \{0.58,0.26,0.53,0.34,0.44\} \\ {\textbf{v}}_3 &{}= \{0.54,0.44,0.34,0.53,0.35\} \end{array} \end{aligned}$$
  Since layers 1 and 2 have the same dependency structure as the prior scenario, the centrality values are unchanged. While the results for layer 3 are not dramatically different relative to the prior example, the increase in the centrality of node 1 in layer 3 is consistent with the fact that it now has a negative association with the smallest centrality node in layer 2.
5. *All layers are dependency case B with cycle* If layer 1 is dependent on layer 3, layer 2 dependent on layer 1 and layer 3 dependent on layer 2, a cycle is introduced in the layer dependency graph, the $\tilde{{\textbf{A}}}$ matrix takes the form:
  $$\begin{aligned} \tilde{{\textbf{A}}} = \begin{bmatrix} 0 &{}\quad 0 &{}\quad 1 \\ 1 &{}\quad 0 &{}\quad 0 \\ 0 &{}\quad 1 &{}\quad 0 \end{bmatrix} \end{aligned}$$
  and the constrained eigenvector centralities are:
  $$\begin{aligned} \begin{array}{rcl} {\textbf{v}}_1 &{}= \{0.40,0.68,0.42,0.38,0.25\}\\ {\textbf{v}}_2 &{}= \{0.58,0.21,0.55,0.36,0.43\} \\ {\textbf{v}}_3 &{}= \{0.48,0.40,0.43,0.52,0.39\} \end{array} \end{aligned}$$

### Random graph example

A more complex example of our constrained multilayer model involves the analysis of interdependent random graphs. Specifically, we simulated a two layer network where each layer was generated as an interconnected group of 5 Erdos–Renyi random graphs with 20 nodes using the igraph R package (Csardi and Nepusz 2006) function call *sample_islands(islands.n=5, islands.size=20, islands.pin=0.2, n.inter=1)*. Constrained eigenvector centrality values were then computed using the proposed algorithm for five different dependency structures:

$$\begin{aligned} \tilde{{\textbf{A}}}_1 = \begin{bmatrix} 1 &{}\quad 0 \\ 0 &{}\quad 1 \\ \end{bmatrix}, \tilde{{\textbf{A}}}_2 = \begin{bmatrix} 0.9 &{}\quad 0.1 \\ 0 &{}\quad 1 \\ \end{bmatrix}, \tilde{{\textbf{A}}}_3 = \begin{bmatrix} 0 &{}\quad 1 \\ 0 &{}\quad 1 \\ \end{bmatrix}, \tilde{{\textbf{A}}}_4 = \begin{bmatrix} 1 &{}\quad 0 \\ 0.1 &{}\quad 0.9 \\ \end{bmatrix} \tilde{{\textbf{A}}}_5 = \begin{bmatrix} 1 &{}\quad 0 \\ 1 &{}\quad 0 \\ \end{bmatrix} \end{aligned}$$

**Fig. 2:**
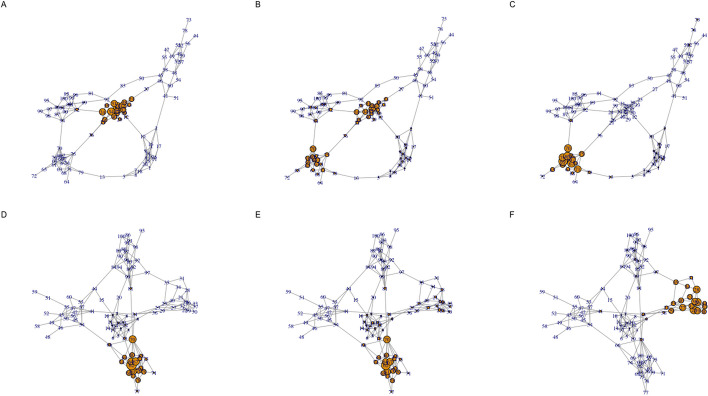
Centrality visualization for a two layer random graph example. Each row corresponds to a separate layer generated as a interconnected group of 5 Erdos–Renyi random graphs that each have 20 nodes. Node size corresponds to eigenvector centrality value. Panels A and D visualize node eigenvector centrality values when the layers are independent. Panels B and E visualize eigenvector centrality values when 10% of the importance of adjacent nodes is based on the other layer, which is kept independent. Panels C and F visualize node eigenvector centrality when adjacent node importance is entirely based on the other layer

**Fig. 3:**
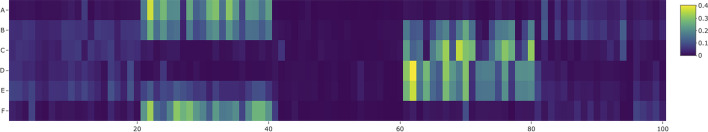
Heatmap visualization of the two layer centrality values shown in Fig. 2. The columns of the heatmap represent network nodes and the rows capture the node centrality values for the network layer in the corresponding panel in Fig. 2

These dependency structures, and the associated results in Fig. 2 have the following interpretations:

- $\tilde{{\textbf{A}}}_1$: This represents dependency case A (as defined in the “Eigenvector centrality for multilayer networks with inter-layer constraints on adjacent node importance” section), i.e., the two layers are completely independent. Panels A and D in Fig. 2 and rows A and D in Fig. 3 visualize the eigenvector centrality values for each layer in this scenario.
- $\tilde{{\textbf{A}}}_2$: This represents a mixture of dependency cases A and C with layer 2 independent and layer 1 having adjacent node importance that is a mixture of 10% layer 2 and 90% layer 1, i.e., close to the independence case. Panels B and D in Fig. 2 and rows B and D in Fig. 3 visualize the corresponding eigenvector centrality values. Interestingly, the small dependence of layer 1 on layer 2 results in a significant shift in eigenvector centrality values but with the distribution of values still dictated by the layer 1 structure.
- $\tilde{{\textbf{A}}}_3$: This represents a mixture of dependency cases A and B with layer 2 independent and adjacent node importance for layer 1 completely based on layer 2. Panels C and D in Fig. 2 and rows C and D in Fig. 3 reflect the eigenvector centrality values for this case. As expected, the centrality values for layer 1 are quite distinct from the independence case seen in panel A but with the overall pattern still constrained by the layer 1 topology.
- $\tilde{{\textbf{A}}}_4$: Similar to $\tilde{{\textbf{A}}}_2$, this is a mixture of dependency cases A and C but with the roles of layer 1 and 2 reversed. Panels A and E in Fig. 2 and rows A and E in Fig. 3 capture the eigenvector centrality values. In this case, there is a less dramatic shift in the dominant eigenvector centralities for layer 2 relative to the independence case.
- $\tilde{{\textbf{A}}}_5$: Similar to $\tilde{{\textbf{A}}}_3$, this is a mixture of dependency cases A and B with layers 1 and 2 reversed. Similar to case 3, the eigenvector centrality values for layer 2 are completely distinct from the independence case while still being constrained by the layer 2 topology. Panels A and F in Fig. 2 and rows A and F in Fig. 3 capture the eigenvector centrality values.

### Random graph example using inter-layer edges

**Fig. 4:**
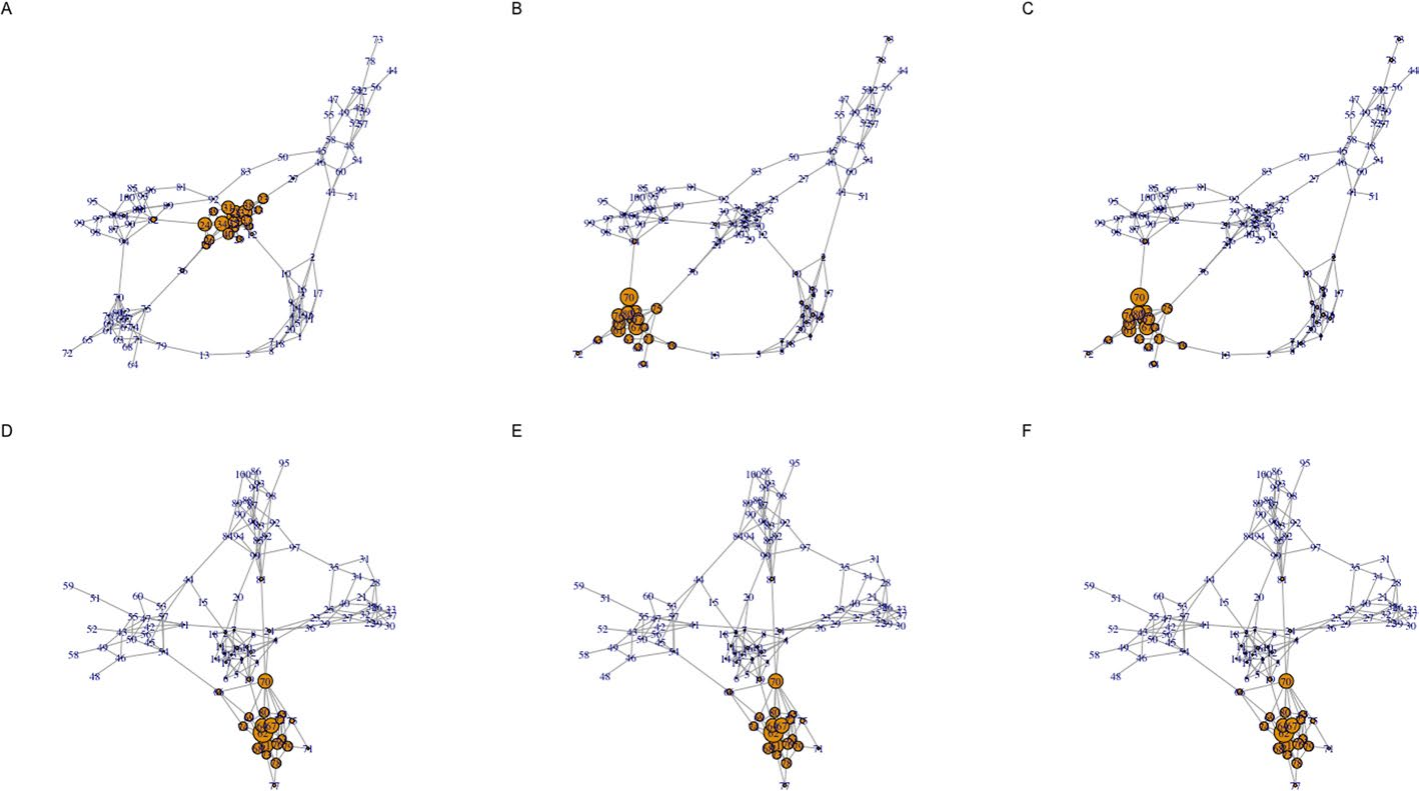
Centrality visualization for a two layer random graph example with inter-layer edges. Each row corresponds to a separate layer generated as a interconnected group of 5 Erdos–Renyi random graphs that each have 20 nodes (the network visualized in Fig. 2 has an identical structure). Similar to Fig. 2, panels A and D visualize node eigenvector centrality values when the layers are independent. Panels B and E visualize eigenvector centrality when directed edges of weight 0.1 are introduced between the nodes in one layer and the nodes in the other layer. Panels C and F visualize eigenvector centrality when directed edges of weight 1 are introduced between the nodes in one layer and the nodes in the other layer

**Fig. 5:**
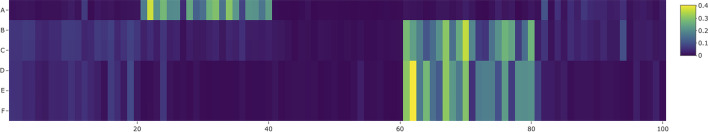
Heatmap visualization of the two layer centrality values shown in Fig. 4. The columns of the heatmap represent network nodes and the rows capture the node centrality values for the network layer in the corresponding panel in Fig. 4

If this random multilayer network is instead analyzed using the standard approach of representing dependencies between layers using inter-layer edges (as detailed in the “Comparison method based on inter-layer edges” section above), a distinct pattern of centrality values are computed for dependency matrices $\tilde{{\textbf{A}}}_2$ and $\tilde{{\textbf{A}}}_4$ as visualized by panels B and E in Fig. 4 and rows B and E in Fig. 5. In this scenario, there is little distinction between panels B and E and panels C and F, which follows from the fact that both result in directed edges from the nodes in one layer to the nodes in the other layer (from layer 1 to 2 in panels B and C and from layer 2 to layer 1 in panels E and F). Interestingly, the centrality values for $\tilde{{\textbf{A}}}_3$ (panel C in Figs. 2, 4) are very similar for both the proposed method and the inter-layer edge approach. By contrast, the two approaches yield dissimilar centrality patterns for $\tilde{{\textbf{A}}}_5$ (panel F in Figs. 2, 4).

### Random graph example using negative dependency weights

**Fig. 6:**
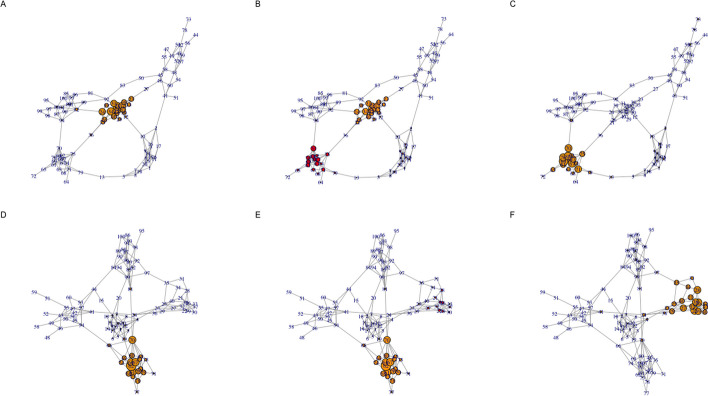
Centrality visualization for a two layer random graph example with negative dependency weights for Panels B and E. Each row corresponds to a separate layer generated as a interconnected group of 5 Erdos–Renyi random graphs that each have 20 nodes (the network visualized in Figs. 2, 4 have identical structure). Similar to Fig. 2, panels A and D visualize node eigenvector centrality values when the layers are independent. Panels B and E visualize eigenvector centrality when one layer is independent and adjacent node importance for one layer is a mixture of a weight 1.1 self dependency and weight $-$0.1 dependency on the other layer. Similar to Fig. 2, Panels C and F visualize node eigenvector centrality when adjacent node importance is entirely based on the other layer

**Fig. 7:**
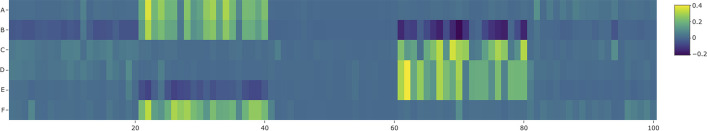
Heatmap visualization of the two layer centrality values shown in Fig. 6. The columns of the heatmap represent network nodes and the rows capture the node centrality values for the network layer in the corresponding panel in Fig. 6

Panels B and E in Fig. 6 and rows B and E in Fig. 7 visualize the centrality values when negative dependency weights are introduced for dependency matrices $\tilde{{\textbf{A}}}_2$ and $\tilde{{\textbf{A}}}_4$. Specifically, the dependency adjacency matrices for these scenarios have been changed to the following:

$$\begin{aligned} \tilde{{\textbf{A}}}_2^* = \begin{bmatrix} 1.1 &{}\quad -\,0.1 \\ 0 &{}\quad 1 \\ \end{bmatrix}, \tilde{{\textbf{A}}}_4^* = \begin{bmatrix} 1 &{}\quad 0 \\ -\,0.1 &{}\quad 1.1 \\ \end{bmatrix} \end{aligned}$$

For Panel B in Fig. 6, the introduction of a negative dependency between layer 1 and layer 2 results in negative centrality values for the lower left island of nodes. By contrast, when the dependency weight is positive, nodes in this island have positive centrality values (as shown in Panel B of Fig. 2). For Panel C in Fig. 6, a similar phenomenon is observed, i.e., the nodes in the island on the right that had increased positive centrality values when a positive weight was used (as shown in Fig. 2) have negative centrality values in this scenario. While these results are consistent with the expected impact of a negative dependency on adjacent node importance, they violate the property of standard eigenvector centrality that all values are positive.

### Simulation analysis

**Fig. 8:**
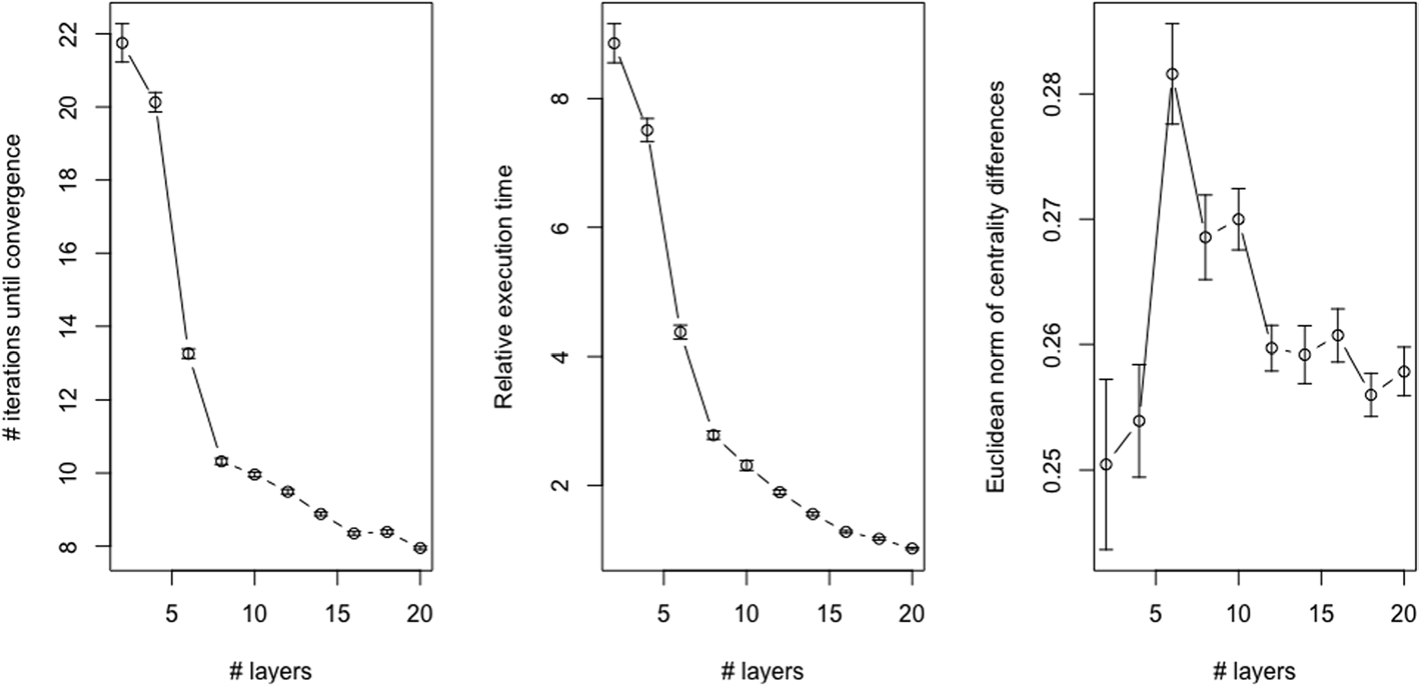
Results on simulated multilayer networks with a fixed number of nodes per layer and varying number of layers (see the “Simulation analysis” section for details on the simulation model). In all panels, the points represent the mean value across 100 simulated networks and error bars capture ± 1 standard error of the mean. The left panel plots the number of iterations of Algorithm 1 until convergence. The middle panel plots the relative execution time for Algorithm 1 versus the inter-layer edge approach. The right panel plots the Euclidean norm of the difference between the centrality values generated by Algorithm 1 and those generated by the inter-layer edge approach

**Fig. 9:**
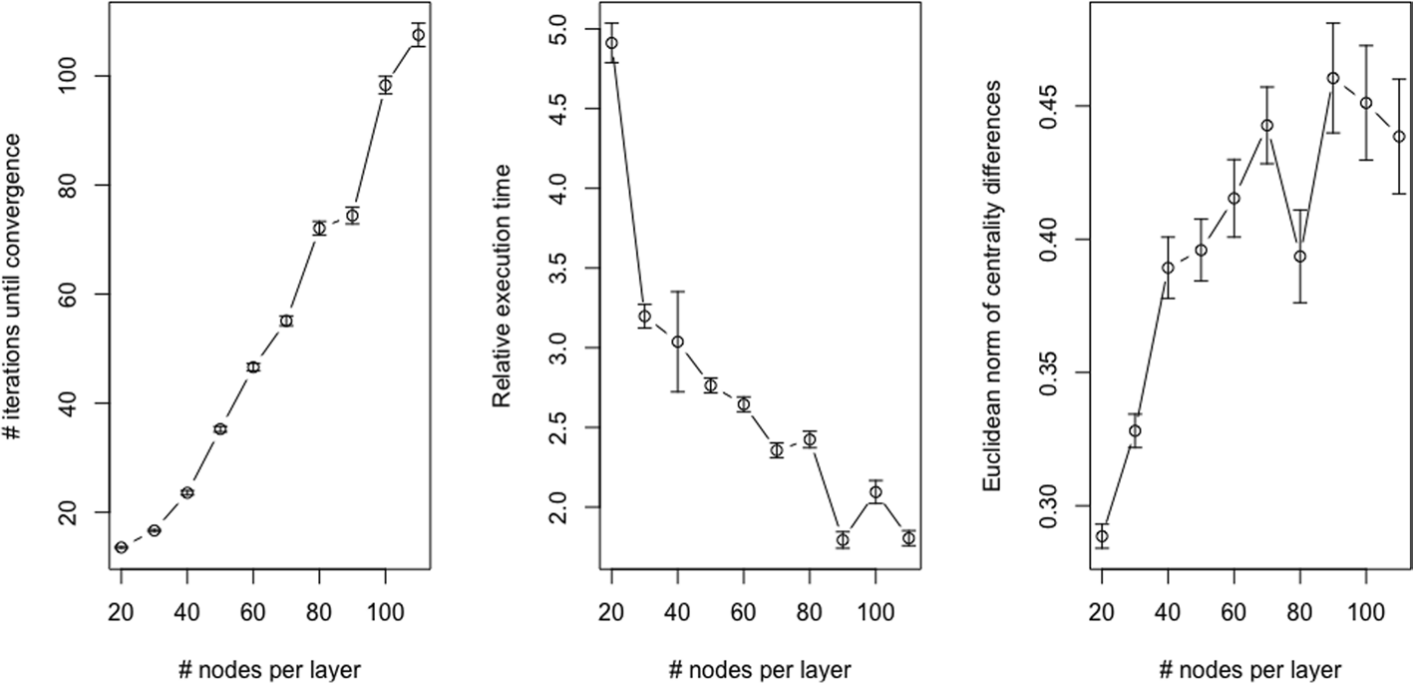
Results on simulated multilayer networks with a fixed number of layers and varying number of nodes per layer (see the “Simulation analysis” section for details on the simulation model). In all panels, the points represent the mean value across 100 simulated networks and error bars capture ± 1 standard error of the mean. The left panel plots the number of iterations of Algorithm 1 until convergence. The middle panel plots the relative execution time for Algorithm 1 versus the inter-layer edge approach. The right panel plots the Euclidean norm of the difference between the centrality values generated by Algorithm 1 and those generated by the inter-layer edge approach

**Fig. 10:**
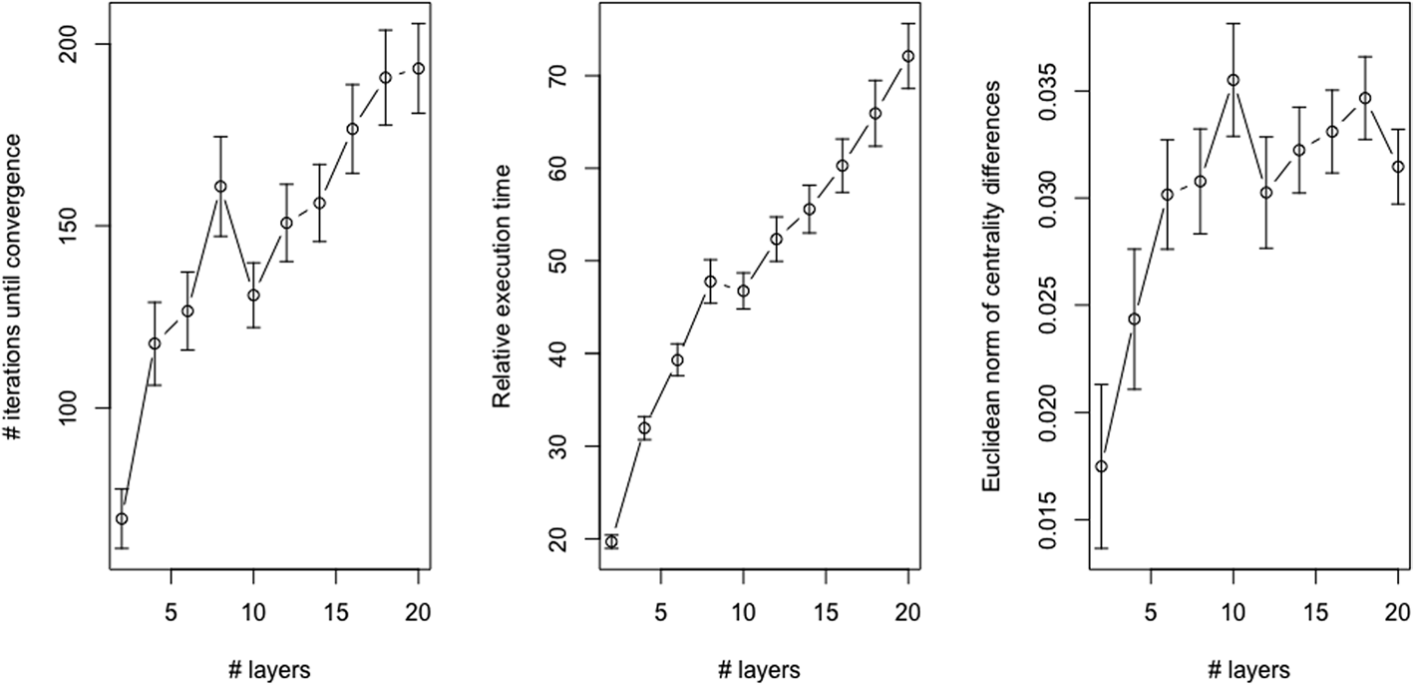
Results on simulated multilayer networks with a fixed number of nodes per layer and varying number of layers (see the “Simulation analysis” section for details on the simulation model). In contrast to the results shown in Figs. 8 and 9, all of the layers in the simulated network are independent. In all panels, the points represent the mean value across 100 simulated networks and error bars capture ± 1 standard error of the mean. The left panel plots the number of iterations of Algorithm 1 until convergence. The middle panel plots the relative execution time for Algorithm 1 versus the inter-layer edge approach. The right panel plots the Euclidean norm of the difference between the centrality values generated by Algorithm 1 and those generated by the inter-layer edge approach

To more thoughly explore the behavior of Algorithm 1, we analyzed random networks with a varying number of layers and number of nodes per layer. Similar to the example random network analyzed in the “Random graph example” section, each layer in the multilayer network was generated as an interconnected group of 5 Erdos–Renyi random graphs that each have the same number of nodes (represented by the variable *x*) using the igraph R package (Csardi and Nepusz 2006) function call *sample_islands(islands.n=5, islands.size=x, islands.pin=0.2, n.inter=1)*. This simulation analysis was executed using three schemes: (1) the number of nodes per layer was fixed at 20 and the number of layers was varied between 2 and 20 (results shown in Fig. 8), (2) the number of layers was fixed at 5 and the number of nodes layer was varied between 20 and 110 (results shown in Fig. 9), and (3) the number of nodes per layer was fixed at 20 and the number of layers was varied between 2 and 20 but the layers were independent (results shown in Fig. 10). For schemes 1 and 2, the dependency structure for each layer was modeled as dependency case C by generating the elements of each row of the inter-layer dependency adjacency matrix ${\tilde{\mathbf {A}}}$ as standard uniform random variables normalized to sum to 1; for scheme 3, ${\tilde{\mathbf {A}}}$ was the identity matrix.

For each combination of number of layers and number of nodes, a total of 100 multilayer networks were simulated and constrained eigenvector centrality values were generated using both Algorithm 1 (requiring per-layer convergence) and the inter-layer edge approach detailed in the “Comparison method based on inter-layer edges” section. For scheme 3, no inter-layer edges were introduced so power iteration was simply applied to each layer separately. Figures 8, 9, and 10 visualize the results of this simulation analysis. Specifically, each figure plots the mean and standard error of the number of iterations until convergence for Algorithm 1 (left panel), the relative execution time between Algorithm 1 and the inter-layer edge approach (middle panel), and the Euclidean norm of the difference between the centrality values computed using the two approaches (right panel). For this last metric, it is important to note that since the Euclidean norm of each vector of centrality values is 1 (i.e., the associated eigenvectors are standardized to length 1), this is equivalent to the ratio of the norm of the differences to the norm of centrality values. The results of this simulation analysis revealed a number of interesting trends:

- *Convergence* As expected, the number of iterations until convergence for Algorithm 1 increases with the number of nodes per layer (left panel of Fig. 9). By contrast, the number of iterations until convergence is inversely correlated with the number of layers in the multilayer network when the layers are dependent (left panel of Fig. 8). This inverse association is due to the dependencies between the pseudo-eigenvalue problems associated with each layer in this scenario. This interpretation is confirmed by the fact that, when all layers are independent, the number of iterations until convergence increases with the number of layers (left panel of Fig. 10). The positive association in this case follows from the fact that per-layer convergence is required so a larger number of layers increases the expected value of the maximum number of iterations. The number of iterations until convergence is also markedly larger in the independence case.
- *Execution time* As expected, the execution time for Algorithm 1, which is simultaneouly applying power iteration to a collection of matrices, is greater than the time required for the inter-layer edge approach, which applies power iteration to just a single matrix (middle panels of Figs. 8, 9). Because the relative performance difference shrinks as both the number of layers and number of nodes increases, the computational cost is likely comparable in practice. As expected, the relative performance gap between the two methods is much larger in the scenario where all of the layers are independent (middle panel of Fig. 10).
- *Centrality value differences* Consistent with the results shown in the “Random graph example” section, the centrality values computed using Algorithm 1 have a relatively large deviation from those computed using the inter-layer edge approach when the layers are dependent, i.e., the norm of the difference between the centrality values is 25–50% of the norm of either set of centrality values (right panel of Figs. 8, 9). By contrast, when the layers are independent, both approaches yield roughly equivalent centrality values (right panel of Fig. 10) with the small deviation due to differences in how the convergence test is evaluated numerically.

## Future directions

Our future work in this area includes exploring the theoretical properties of our multilayer network model and interleaved power iteration algorithm (e.g., proof of convergence), exploring the theoretical and practical implications of negative inter-layer dependency weights, and applying our technique to real multilayer networks including a network model for the cell signaling problem described above that is created using tissue imaging and genomic profiling data.

## Data Availability

An implementation of Algorithm 1 and the comparison inter-layer edge technique described in the “Comparison method based on inter-layer edges” section are available via the CMLC (Constrained Multilayer Centrality) R package. The CMLC R package along with example vignettes that generate the results in the “Simple example” to “Simulation analysis” sections are available at https://hrfrost.host.dartmouth.edu/CMLC/.
